# Foot Placement Characteristics and Plantar Pressure Distribution Patterns during Stepping on Ground in Neonates

**DOI:** 10.3389/fphys.2017.00784

**Published:** 2017-10-10

**Authors:** F. Sylos-Labini, S. Magnani, G. Cappellini, V. La Scaleia, A. Fabiano, S. Picone, P. Paolillo, A. Di Paolo, F. Lacquaniti, Y. Ivanenko

**Affiliations:** ^1^Center of Space BioMedicine, University of Rome Tor Vergata, Rome, Italy; ^2^Neuromotor Physiology Laboratory, Fondazione Santa Lucia (IRCCS), Rome, Italy; ^3^Department of Computer, Control and Management Engineering, Sapienza University of Rome, Rome, Italy; ^4^Neonatology and Neonatal Intensive Care Unit, Casilino Hospital, Rome, Italy; ^5^Neonatology and Neonatal Intensive Care Unit, Ospedale San Giovanni, Rome, Italy; ^6^Department of Systems Medicine, University of Rome Tor Vergata, Rome, Italy

**Keywords:** neonates, stepping pattern, limb loading, foot pressures, kinematics, EMG activity, early development, human locomotion

## Abstract

Stepping on ground can be evoked in human neonates, though it is rather irregular and stereotyped heel-to-toe roll-over pattern is lacking. Such investigations can provide insights into the role of contact- or load-related proprioceptive feedback during early development of locomotion. However, the detailed characteristics of foot placements and their association with motor patterns are still incompletely documented. We elicited stepping in 33 neonates supported on a table. Unilateral limb kinematics, bilateral plantar pressure distribution and EMG activity from up to 11 ipsilateral leg muscles were recorded. Foot placement characteristics in neonates showed a wide variation. In ~25% of steps, the swinging foot stepped onto the contralateral foot due to generally small step width. In the remaining steps with separate foot placements, the stance phase could start with forefoot (28%), midfoot (47%), or heel (25%) touchdowns. Despite forefoot or heel initial contacts, the kinematic and loading patterns markedly differed relatively to toe-walking or adult-like two-peaked vertical force profile. Furthermore, while the general stepping parameters (cycle duration, step length, range of motion of proximal joints) were similar, the initial foot contact was consistently associated with specific center-of-pressure excursion, range of motion in the ankle joint, and the center-of-activity of extensor muscles (being shifted by ~5% of cycle toward the end of stance in the “heel” relative to “forefoot” condition). In sum, we found a variety of footfall patterns in conjunction with associated changes in motor patterns. These findings suggest the potential contribution of load-related proprioceptive feedback and/or the expression of variations in the locomotor program already during early manifestations of stepping on ground in human babies.

## Introduction

Different types of stepping-like responses can be evoked in human neonates. Visual stimulation can elicit stepping-like leg movements in the air, whose features depend on the specific characteristics of the visual stimuli (Barbu-Roth et al., 2014). Stepping on ground can be elicited provided the infant's body weight is adequately supported. Here we are concerned exclusively with stepping on ground.This typically involves alternating limb movements at relatively slow speeds and exaggerated foot lift, and it is rather irregular. Other substantial differences relative to mature gait are excessive co-contraction of many leg muscles (Forssberg, 1985; Okamoto et al., 2001; Dominici et al., 2011; Teulier et al., 2012) and co-activation of motoneuron pools at both lumbar and sacral levels of the spinal cord (Ivanenko et al., 2013). During stepping, posture is flexed and stereotyped heel-to-toe roll-over pattern is lacking. The spatiotemporal dynamics of motoneuron activation in the lumbosacral spinal cord becomes more differentiated and the separation of activity becomes more prominent as a child grows. Functional sensory-motor circuitry, adult-like footfall pattern and transition to coordinated activity of motoneurons emerge with specific timing during development (Thelen and Cooke, 1987; Lacquaniti et al., 2012; Yang et al., 2015).

The lack of heel-to-toe roll-over pattern is a characteristic feature of neonatal stepping. Furthermore, newborns typically exert vertical forces supporting part (~30–40%) of their weight. Nevertheless, weight support through the feet and extension of the legs at the end of stance are important proprioceptive inputs to initiate stepping in infants (Yang et al., 2004). In addition, tactile foot stimulation may evoke reflex responses, e.g., stimulation of the sole of the foot may induce dorsiflexion of the big toe along with a brisk flexion of the whole limb, the Babinski sign (Babinski, 1922), and the flexion reflex responses to innocuous stimulation are already present in neonates (Andrews and Fitzgerald, 1999). The contribution of proprioception in the specific planar surface areas and the role of foot-function in the immature neonatal stepping still remain unclear.

Some differences between infants and adults in foot function and proprioceptive feedback might be expected because of morphologic changes. The ossification of the foot skeleton and development of the bony structure of the longitudinal arch only starts ~1 year after birth and continues up to the age of 5 years (Straus, 1926; Maier, 1961). A fat pad underneath the foot plantar surface protects the fragile cartilaginous tissue of the foot skeleton and also affects the loading area of the plantar surface during stepping. Stepping in older (>6 mo) infants is surprisingly adaptable to a range of tactile inputs (Yang et al., 2004), however, adaptability of neonatal stepping is less clear. Studies in older infants suggest that a child's foot goes through significant changes in shape and loading characteristics, especially when the child starts to stand and walk independently (Bertsch et al., 2004; Hallemans et al., 2006). In particular, they showed large intra-individual variations and a trend from the flat foot contact to heel-to-toe roll-over with increasing walking experience. How do these foot-function characteristics compare to the more immature neonatal stepping?

While the variable footfall pattern in neonates was noticed in many studies, the detailed characteristics of foot placements, plantar pressure distribution and their association with motor patterns were not systematically investigated for neonatal stepping. However, in his pioneering study, Forssberg (1985) remarked that newborns typically hit the supporting surface with the lateral aspect of the foot sole, heel and toes contacting ground at about the same time, but occasionally the heel contacted ground first. He further remarked that the typical adult-like pattern, consisting of a real heel strike in front of the body, could never be observed in newborns. Since plantar surface plays an important role in proprioception, such studies may also provide insights into maturation and contribution of proprioceptive feedback during early development of locomotion. Therefore, our primary objective was to quantify the footfall and foot loading patterns in stepping neonates.

## Materials and methods

### Participants

We elicited stepping in 33 full-term neonates (Apgar score >7 at 1 and 5 min, uneventful delivery and perinatal history) supported on a table. They were 3.8 ± 4.0 days old (mean ± SD), their body weights ranged from 2.15 to 4.09 kg (3.2 ± 0.5 kg) and body (crown-to-heel) length from 44 to 57 cm (50.6 ± 2.4 cm). Neonate stepping was recorded at the well-baby maternity ward, after obtaining informed consent from the parents. The protocol had been previously approved by the relevant Ethics Committees (protocol CEI/15843 approved by the Ethics Committee of Roma Asl RMC, 9.03.2009, further approved by the General Director of Regione Lazio on 1.07.2009, document n. 609), and was conducted in accordance with the Declaration of Helsinki for experiments on humans.

### Data recording

A table (~80 × 110 × 80 cm) was placed within the testing area, covered with a walkway measuring load distribution underneath the plantar surface of the foot, so that the infant stepped along a 1 m walkway. The environment in which the experiments took place (a warm, quiet and dimly lit room at the hospital nursery) was appropriate for the neonates. To elicit stepping, infants were held upright under the armpits (with the head supported in midline) with their feet touching the horizontal flat walkway surface. The infant was slightly tilted forwards in order to facilitate the stepping response. Four different pediatricians performed these experiments in 10, 7, 9, and 7 neonates, respectively (33 neonates total). Elicitation of stepping responses was typically successful when the child was sufficiently aroused (Thelen et al., 1982). No movement recording was carried out if the infant was drowsy or asleep. The infants were allowed to support as much of their own weight as possible, the rest being supported by the pediatrician holding the infant. The duration of the whole experiment (including marker and EMG electrode placements) was <30 min. Short trials, depending on the child's endurance, were recorded with rest breaks in between.

**Figure 1:**
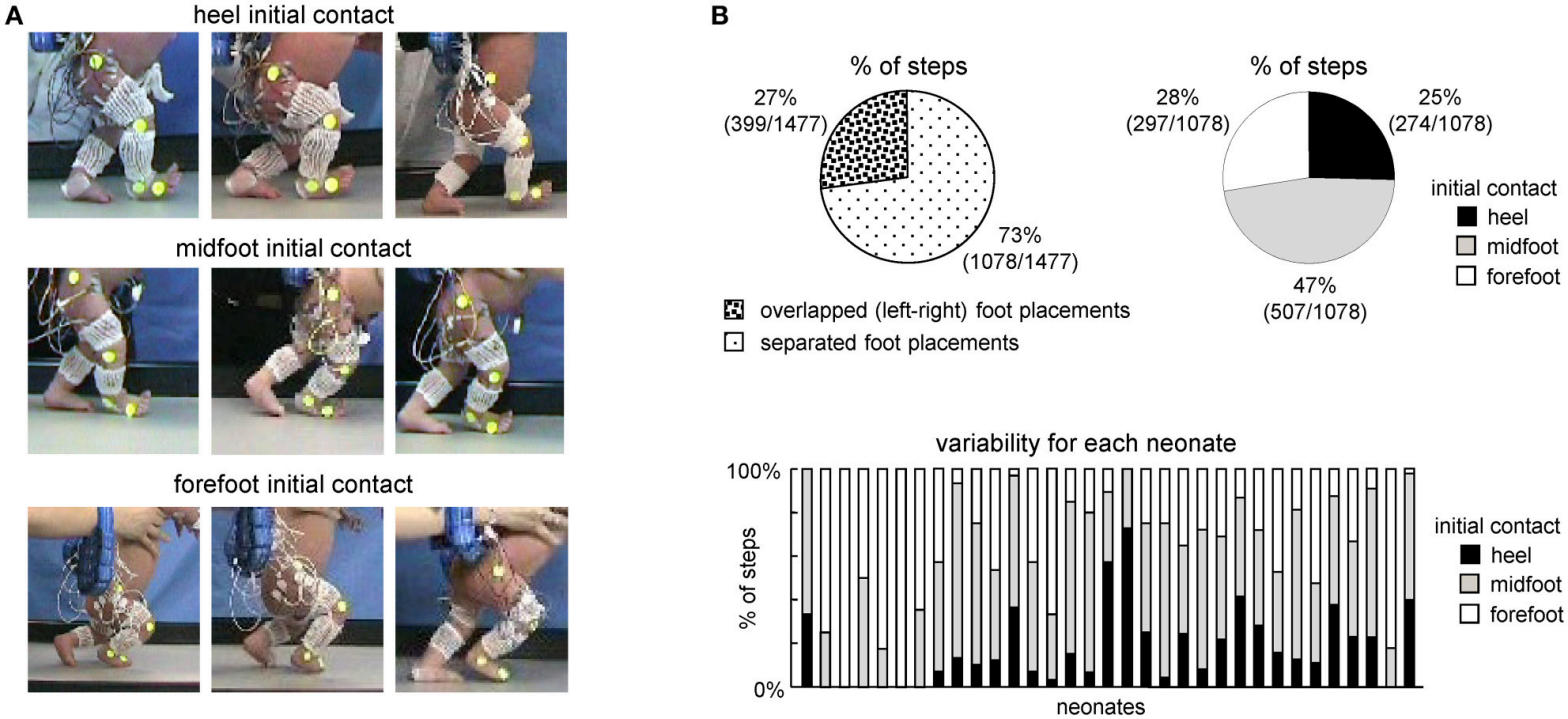
General foot placement characteristics during stepping in neonates. **(A)** Examples of heel, midfoot and forefoot initial touchdowns in nine neonates. **(B)** Percent of steps with overlapped foot placements when one leg crossed the other (left) and with different types of touchdown contacts in neonates (right). In the latter case (right panel), only separated (not overlapped) steps were analyzed. On the bottom–percentage of steps with different initial foot contacts for each child.

Unilateral limb kinematics, bilateral foot pressure and EMG activity were recorded, with all recordings being synchronized. Kinematics of the right side of the baby in the sagittal plane was recorded at 25 frames per second by a video camera (Canon MD160, Canon Inc., Japan). Four markers (12 mm diameter) were attached to the skin over the hip (greater trochanter, GT), knee (lateral femur epicondyle, LE), ankle (lateral malleolus, LM), and fifth metatarsophalangeal joint (5MT) of the right leg (Figure 1A). Plantar pressure distribution patterns were measured at 50 Hz by the Walkway Tekscan (Tekscan Inc, USA, 44 × 95-cm, 4 sensors/cm^2^, factory-calibrated for the low pressure values in infants).

EMG activities were recorded bilaterally at 2 kHz from up to 22 muscles simultaneously using the wireless Zerowire system (Aurion Srl, Italy) with miniature (2-mm diameter) electrodes, bandwidth of 20–1,000 Hz with an overall gain of 1,000. The skin was cleaned and rubbed slightly with alcohol before placing the electrodes. Generally, we used miniature (2 mm recording diameter, to minimize cross talk), Ag-AgCl, reusable, surface EMG disc electrodes (Beckman Instruments). In few instances (based on the pediatrician's recommendation), we used disposable surface electrodes (15 mm; Ambu). To minimize potential movement artifacts, preamplified EMG sensor units were attached on the experimenter wrist, and twisted pairs of wires (between electrodes and units) were limited to 25 cm length and fixed along the infant leg using elastic gauze (Figure 1A). During the rest periods (1–2 min) between trials, EMGs were often monitored, to determine whether clear and separate bursts of EMG could be obtained from each muscle group. Quiet periods during the sequence of recording allowed us to estimate clean baselines and the noise level in each channel. By recording slightly different sets of muscles in different infants we obtained the whole data set of 22 bilateral EMGs (each muscle was recorded at least in 10 neonates: range 10–30 neonates for each muscle assessed). The recorded muscles were: gluteus maximus (GM), tensor fascia latae (TFL), adductor longus (Add), vastus lateralis (VL), vastus medialis (VM), rectus femoris (RF), hamstring (HS), tibialis anterior (TA), gastrocnemius lateralis (LG), gastrocnemius medialis (MG), and soleus (Sol).

### Classification of steps

Successful sequences of stepping were identified off-line from video recordings. In particular, we used the same criteria for choosing the steps as in previous studies (Yang et al., 1998; Ivanenko et al., 2013), and consisted of at least two consecutive alternating strides of both legs. Gait initiation and termination steps were excluded from the analysis. Given the limited length of the walkway (110 cm, see above), on average, we recorded and analyzed 3.3 ± 1.4 consecutive alternating steps. We did not analyse isolated or other types of steps that did not involve consecutive alternation. Classification of the initial foot contact (forefoot, midfoot or heel contact, see examples in Figure 1, left) was determined independently by visual inspection of video recordings by two different experimenters. Normally, the judgments of the two experimenters coincided for the definition of the heel and forefoot touchdown contacts. In some cases, one of the experimenters judged them as midfoot and in such cases we classified them midfoot. On average, we recorded 16 ± 22 (range 2–124) successful strides per infant (which corresponds to the range of 4–248 steps per infant, since we recorded and analyzed the foot loading data for both right and left legs).Overall, 1477 steps in 33 neonates were analyzed.

### Kinematic data analysis

From the kinematic recordings, the gait cycle was defined as the time between two successive foot touchdown events of the right leg. Touchdown and lift off events were determined by visual inspection of video recordings and confirmed by the foot loading data (if one leg did not cross the other during a stepping movement). The kinematic data were time interpolated over individual gait cycles to fit a normalized 200-point time base for averaging across strides. The following kinematic parameters were calculated: stepping speed (using the GT marker), stride length, stride width, range of angular motion (ROM), foot (5MT marker) lift and limb segment angles at touchdown and lift off. The limb angle was defined as GT–LM. The stride length and foot trajectory were normalized to the limb length (L, determined by summing lengths of the thigh and shank segments) of the infants.

The intersegmental coordination of the thigh, shank, and foot elevation angles in the sagittal plane was evaluated as previously described using the principal component analysis (Borghese et al., 1996; Lacquaniti et al., 2002; Ivanenko et al., 2008). Briefly, the procedure was the following. We computed the covariance matrix of the ensemble of time- varying elevation angles (after subtraction of their mean value) over each gait cycle. The three eigenvectors u_1_–u_3_, rank ordered on the basis of the corresponding eigenvalues, correspond to the orthogonal directions of maximum variance in the sample scatter. The first two eigenvectors u_1_-u_2_ lie on the best-fitting plane of angular covariance and the third eigenvector (u_3_) is the normal to the plane and defines the plane orientation. The planarity of the trajectories was quantified by the percentage of total variation (PV) accounted for by the first two eigenvectors of the data covariance matrix: the closer is PV_1_+PV_2_ to 100% (or the closer is PV_3_ to 0), the smaller the deviation from planarity.

In addition to PV, we assessed orientation and variability of the normal to the covariance plane across steps by calculating u_3_ (the direction cosines with the three axes) and spherical contour of the density distribution of u_3_ axes, using the algorithm proposed by Vollmer (1995) and based on the modified Kamb method (Kamb, 1959). In summary, if *n* points are selected randomly from a uniform population distributed over an area *A*, the probability that any given point will lie within an arbitrary subarea a of A is *p* = *a*/*A*. The number of points occurring within area *a* can be considered as a binomial random variable (which mean is *μ* = *np* and standard deviation is $\sigma =\sqrt{np\cdot \left(1-p\right)}$) with an expected count *E*, equal to the mean *μ*. Kamb (1959) selected a binomial probability model with *E* = *μ* = 3σ so that, given a random sample from a uniform population, the counting circle would be large enough so the observed counts would not be likely to fluctuate wildly from the expected count. Contour levels >3σ (*E*) indicate a density higher than expected for a uniform distribution, and levels <3σ indicate a density lower than expected. The 5σ contour, for example, represents densities 2SDs more than expected (E + 2σ). In the algorithm, the nodes of a regular square (30 × 30) grid are back-projected onto the sphere using stereographic projection. For each node on the sphere the number of data points that fall within a spherical cap of area *a* = 2*π* · (1 − cos *θ*), where *θ* is the semi-apical angle of the cap, are counted with an exponential weighting function in order to smooth the contour. For axial data distributed on a unit hemisphere of area 2*π*, the angle *θ* can be calculated considering that *p* = *a*/*A* = 1 − cos *θ*. The contour levels are drawn by linear interpolation through the grid. A primary benefit of Kamb's method is that it reduces the influence of sample size by separating the counting element size (Vollmer, 1995).

### Foot loading and plantar pressure distribution

**Figure 2:**
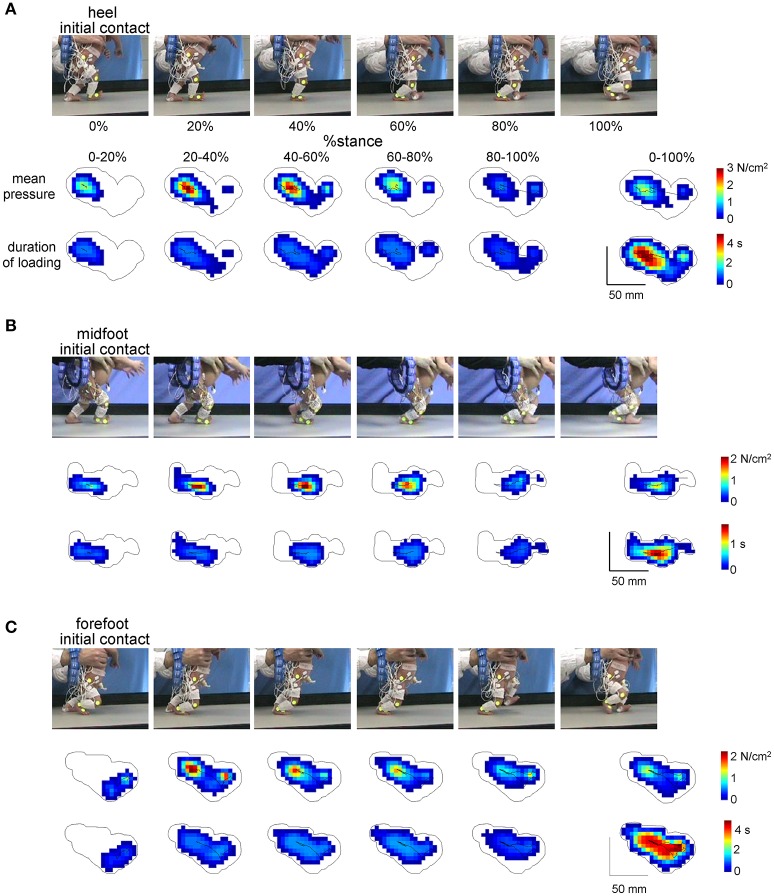
Examples of plantar pressure recordings of different foot-contact patterns (**A**, initial heel contact, **B**, midfoot contact, and **C**, forefoot contact) in three neonates. For each panel from top to bottom: illustration of the stance phase of the right leg in a neonate, corresponding mean pressure values and mean duration of loading of the individual sensors over five intervals of the stance phase (0–20%, 20–40%, 40–60%, 60–80%, and 80–100%) and over the whole stance (0–100%). The trajectories of the center of pressure are also shown for each footprint. Contours around color footprints correspond to the area of all sensors loaded during the whole stance.

For illustrative purposes, to characterize the general pattern of foot pressure distribution, mean pressure values of the individual sensors and mean duration of their loading over the stance phase were displayed using a color scale (Figure 2). From the vertical contact force recordings we calculated the total limb loading (as a sum of forces of individual foot pressure sensors underneath the plantar surface of the foot) and the center of pressure (CoP) throughout the stance phase. They were time interpolated over individual cycles to fit a normalized 200-point time base for averaging. The CoP was normalized to the footprint length. The mean and maximum values of foot loading during stance were calculated for each step. Since the general profile of foot loading in neonates was typically bell-shaped (see Results), we also calculated the timing of its peak. Percent of body weight supported by the experimenter (BWS) was evaluated as (body weight-peak of the foot loading) /body weight.

### EMG data analysis

EMG data from the right leg were high-pass filtered (60 Hz), rectified, low-pass filtered (zero-lag fourth-order Butterworth filter with cutoff at 5 Hz) and time interpolated over individual gait cycles to fit a normalized 200-point time base for averaging across strides. In addition to computing the ensemble-averaged EMG waveforms and mean EMG activity, we calculated for each muscle the center-of-activity (CoA) throughout the step cycle using circular statistics (“circ_mean.m” function in the CircStat Matlab toolbox, Berens, 2009), as the angle of the vector (first trigonometric moment) which points to the center of mass of that circular distribution using the following formulas:

(1) $$\begin{array}{l} A=\sum_{t=1}^{200}\left(\cos\theta_{t}\times EMG_{t}\right) \end{array}$$

(2) $$\begin{array}{l} B=\sum_{t=1}^{200}\left(\sin\theta_{t}\times EMG_{t}\right) \end{array}$$

(3) $$\begin{array}{l} CoA=\tan^{-1}\left(B/A\right) \end{array}$$

The CoA has been previously used to characterize the overall temporal shifts of EMG or motoneuron activity (Yakovenko et al., 2002; Martino et al., 2014; Sylos-Labini et al., 2014; Cappellini et al., 2016) and was chosen because it was impractical to reliably identify a single peak of activity in the majority of muscles.

### Statistics

Descriptive statistics included the calculation of the mean and SD. Differences in the stepping characteristics across conditions (heel, midfoot, forefoot) were evaluated using one way ANOVA test. *Post-hoc* tests and multiple comparisons analysis were performed by means of the Tukey's Honestly Significant Difference (HSD) test. A Pearson correlation coefficient was used to analyze the relationship between the foot angle at the first and second touchdowns of the same stride. Circular statistics on directional data (Batschelet, 1981; Fisher, 1995) were used to characterize the mean CoA for each muscle and its variability across strides (angular SD). The Watson-William test was used for circular data (CoA) to evaluate the effect of condition. Reported results are considered significant for *p* < 0.05.

## Results

### General foot placement characteristics

The examples of initial touchdowns in different neonates are illustrated in Figure 1A. Foot placement characteristics in neonates showed a wide inter-step and inter-individual variation. In about 25% of steps (399 out of 1477 steps in 33 neonates), one leg crossed the other during the swing phase and the foot touched and stepped onto the contralateral foot (overlapped foot placements, Figure 1B, left panel). In remaining 75% of separated foot placements (1078 steps), the initial foot contact at touchdown occurred at heel (25%), midfoot (47%), and forefoot (28%) (Figure 1B, right panel). In is also worth noting that while most infants displayed a prevalence of a specific initial foot contact across steps, nevertheless, they could produce various foot contacts at touchdown. The percentage of steps with different initial foot contacts for each child is shown in the bottom panel of Figure 1B.

### Foot loading characteristics

Figure 2 illustrates the examples of foot pressure patterns during steps with different initial contacts. Both mean pressure and loading duration footprints at different intervals of the stance phase (every 20% of stance) show that the forefoot, midfoot and hindfoot regions are loaded in all initial foot contact conditions. For instance, even though the initial contact occurred at forefoot, other regions of the foot were often loaded later in stance (Figure 2C), which makes a difference from toe-walking (forefoot loading throughout the whole stance). The total contact area during the stance phase is indicated by the contour. A similar (though in an opposite way) shift of the loaded region occurred in the steps with the initial heel contact: first, the heel region was loaded and then the other regions later on (Figure 2A).

**Figure 3:**
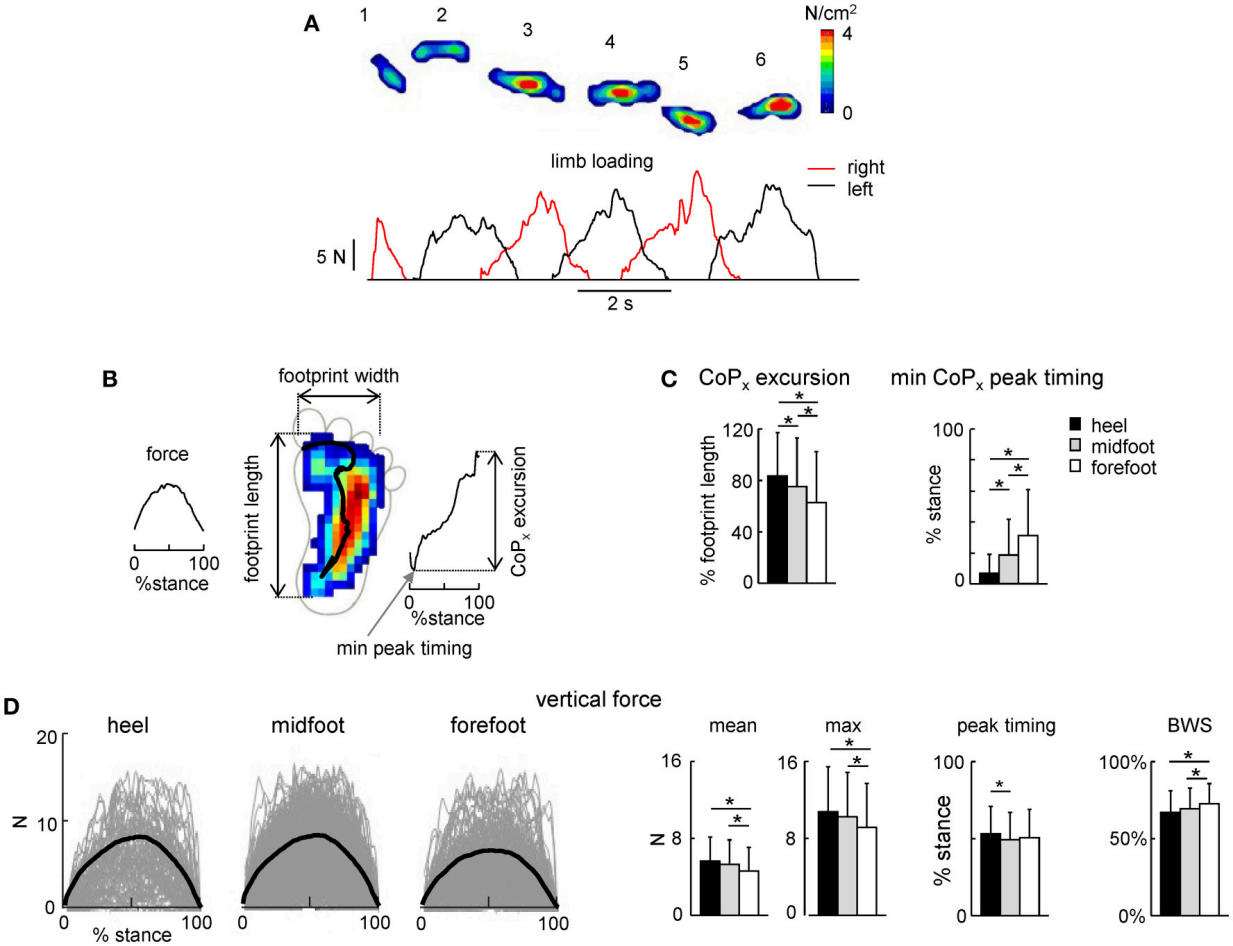
Foot loading characteristics. **(A)** Example of maximal plantar pressure distribution and corresponding limb loading in one neonate across six consecutive steps. **(B)** Example of maximal plantar pressure distribution pattern and CoP displacement during one stride (with the initial heel contact) and the parameters calculated: vertical foot loading force, footprint length and width, CoP excursion and the timing of its minimum peak during stance. **(C)** CoP_x_ excursion and the timing of the min CoP_x_ peak (mean+SD, all strides with a specific initial foot contact were pooled together). CoP_x_ for each child was normalized to the maximum footprint length across all steps. **(D)** Vertical foot loading force and its characteristics (mean, maximum the timing of the maximum force, and percent of body weight support BWS). Black thick curves correspond to the ensemble-averaged (across all steps in all neonates) waveforms vs. normalized stance. Asterisks denote significant differences. Note a generally bell-shaped trajectory of the vertical foot loading with a peak in midstance **(A,D)**.

Local loadings of the plantar side of the foot and CoP displacements were variable for different infants and between different steps of the same individual (Figure 3A). We did not find any significant side difference in the ground force parameters, and therefore we pooled the data for the right and left legs together. Figure 3C shows the general characteristics of the anterior-posterior CoP displacements. Since the total contact area during steps with the forefoot touchdown typically included all three regions of the foot (heel, midfoot and forefoot), the center of pressure was located initially at the forefoot, then shifted posterior, to end again in the forefoot region. Accordingly, the timing of the CoP_x_ minimum was significantly later for the forefoot condition [ANOVA *F*_(2, 1075)_ = 61.9 *p* < 0.00001, Tukey tests *p* ≤ 0.00002, Figure 3C, right panel]. The CoP excursion was nevertheless smaller for the forefoot condition [ANOVA *F*_(2, 1075)_ = 36.6 *p* < 0.00001, Tukey tests *p* ≤ 0.001, Figure 3C, left panel].

The time course of the net vertical ground reaction force is plotted in Figure 3D. Interestingly, in contrast to the two-peaked profile in adults (one peak at the onset and another one at the end of stance, Ivanenko et al., 2002), neonates typically displayed a bell-shaped trajectory of the vertical foot loading (Figures 3A,D) with a peak at midstance for all three initial foot contact conditions (Figure 3D, right panel). The mean and maximum value of the net vertical ground reaction force and its peak timing were slightly though significantly larger for the heel contact steps (Figure 3D).

Percent of body weight supported by the experimenter was about 70% [evaluated as (body weight-peak of the foot loading) / body weight] and slightly larger for the forefoot contact steps (Figure 3D, right panel), consistent with slightly smaller vertical ground reaction force applied by the child in this condition. Four different pediatricians evoked stepping movements in neonates, nevertheless, all three types of the initial foot contact were always present and the mean levels of the BWS were similar for the steps of neonates supported by different testers (61 ± 15%, 70 ± 12%, 73 ± 11%, and 75 ± 13% for each pediatrician, respectively).

### Kinematics of stepping movements

**Figure 4:**
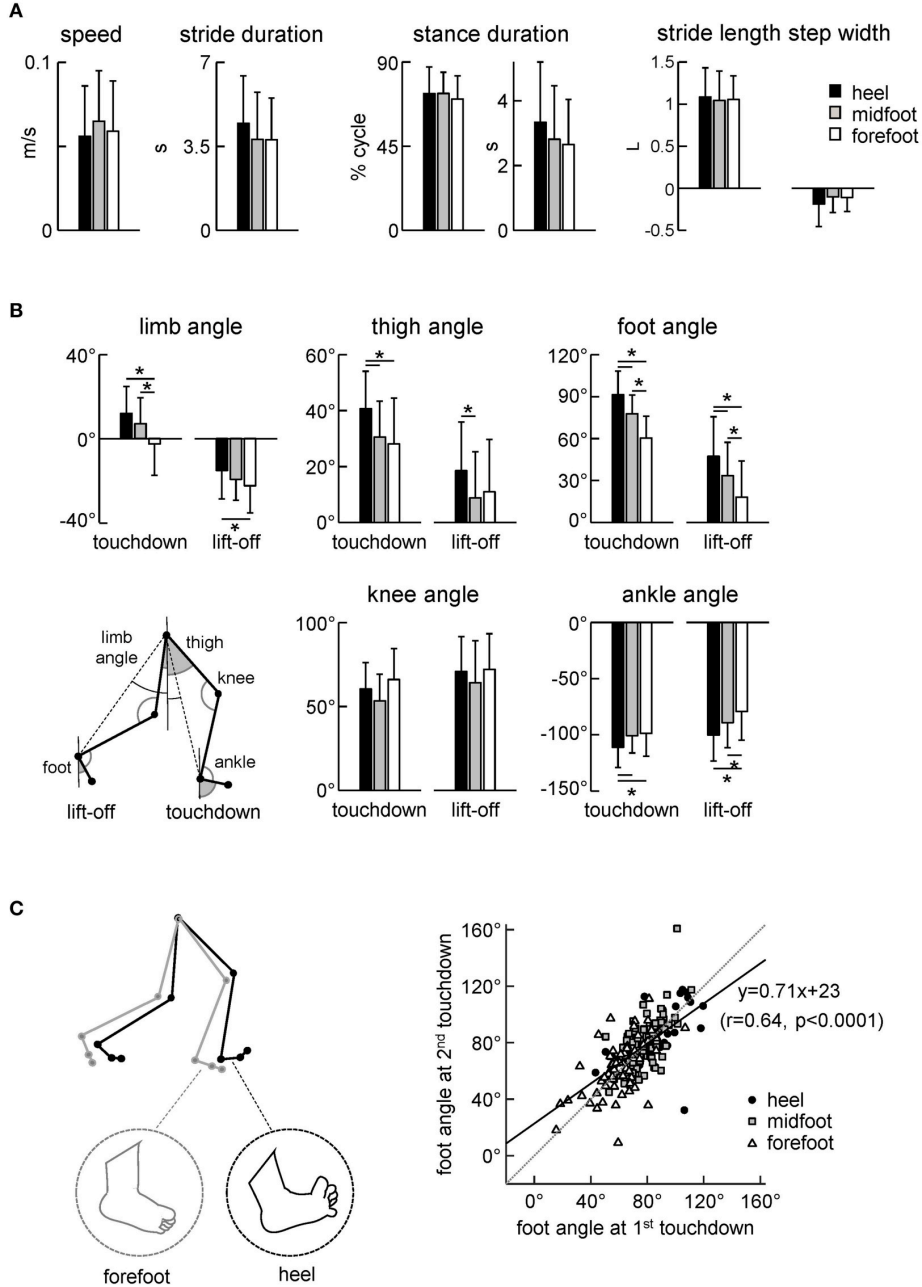
Kinematic stepping parameters. **(A)** Stepping speed, stride duration, stance duration and stride length and width. The stride length was normalized to the lower limb length L. **(B)** Limb angle (positive in the forward direction, i.e., when the distal marker is placed anteriorly to the proximal one), thigh and foot elevation angles and knee and ankle joint angles at touchdown and lift-off. The limb angle was defined as GT-LM (see insert). **(C)** Correlation between the foot angle at the first (0% cycle) and second (100% cycle) touchdown of the same step. Linear regression line with corresponding r and *P*-values are reported. Note a higher correlation of the foot angle between two consecutive touchdowns. The schematic plot on the left illustrates the major kinematic differences between strides with initial heel and forefoot contacts (larger limb angle at touchdown, dorsiflexed ankle joint and dorsiflexed toes for the heel condition). Asterisks denote significant differences between conditions.

We analyzed the kinematics of the strides of the right leg (we did not record the left leg kinematics) that initiated the stance with a specific initial foot contact (heel, midfoot or forefoot). The variable footfall pattern in neonates was not associated with specific changes in the general characteristics of stepping since the cycle duration, stance duration and stride length and width did not significantly differ across conditions (Figure 4A). Consistent with no changes in the stride length [ANOVA: *F*_(2, 186)_ = 0.185 *p* = 0.83] and duration [*F*_(2, 187)_ = 1.55 *p* = 0.22], the stepping speed also did not differ between conditions (0.056 ± 0.03 m/s for heel contact, 0.065 ± 0.03 m/s for midfoot contact, 0.059 ± 0.03 m/s for forefoot contact, *F*_(2, 183)_ = 1.19 *p* = 0.31, Figure 4A left panel). It is also worth noting that even for separated (not overlapped) foot placements the step width was usually small in neonates (even negative, Figure 4A, right panel), in sharp contrast with older children and adults (Ivanenko et al., 2005).

Despite similar general gait parameters (Figure 4A), there were significant differences across conditions in the orientation of the lower limb segments at touchdown and lift-off (Figure 4B). In particular, the limb (hip-ankle), thigh, foot and ankle joint angles at touchdown and lift-off systematically decreased for the heel-midfoot-forefoot conditions [ANOVA for limb angle at touchdown: *F*_(2, 185)_ = 14.5 *p* < 0.00001, Tukey test *p* ≤ 0.00005; limb angle at lift-off: *F*_(2, 184)_ = 3.8 *p* = 0.02, Tukey *p* = 0.02; thigh angle at touchdown: *F*_(2, 184)_ = 7.6 *p* = 0.0007, Tukey *p* ≤ 0.003; thigh angle at lift-off: *F*_(2, 183)_ = 3.3 *p* = 0.04, Tukey *p* = 0.03; foot angle at touchdown: *F*_(2, 186)_ = 46.8 *p* < 0.00001, Tukey *p* ≤ 0.0001; foot angle at lift-off: *F*_(2, 187)_ = 13.4 *p* < 0.00001, Tukey *p* ≤ 0.03; ankle angle at touchdown: *F*_(2, 185)_ = 4.8 *p* = 0.009, Tukey *p* ≤ 0.02; ankle angle at lift-off: *F*_(2, 186)_ = 7.5 *p* = 0.0007, Tukey *p* ≤ 0.04]. The schematic subplot in Figure 4C illustrates major kinematic differences between strides with initial heel and forefoot contacts: larger limb angle at touchdown, dorsiflexed ankle joint and dorsiflexed toes (noted by visual inspection of video recordings) in the initial heel contact condition.

We used the initial foot contact in the gait cycle (0% of cycle) to classify the strides and to characterize foot loading during the stance phase (Figures 2, 3) and the corresponding limb kinematics (Figure 4). Note, however, a higher correlation between the foot angles at two consecutive touchdowns (Figure 4C. right panel) suggesting that the second foot contact tends to be similar to the first one and that the second touchdown (after the swing phase) could likely also be used to classify the preceding stance. Still, for step classification and the analysis of foot loading we used the first foot contact during stance, since the second touchdown occurred only after the swing phase.

**Figure 5:**
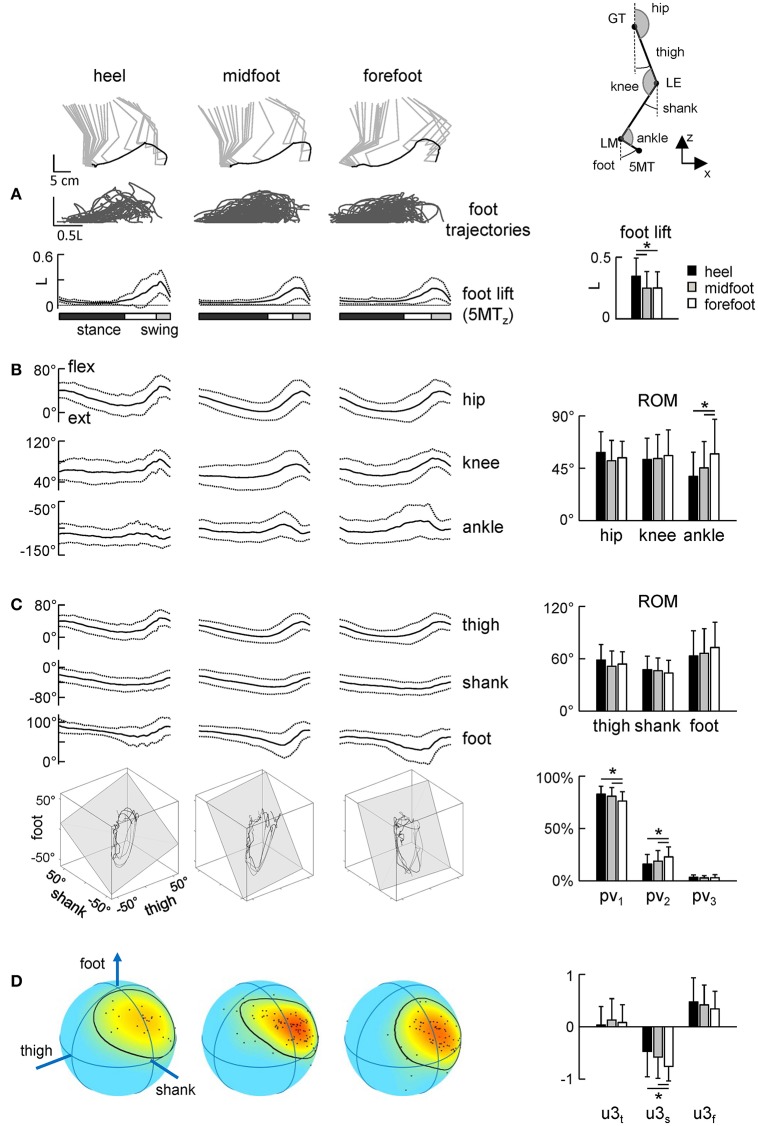
Foot trajectories **(A)** and angular movement characteristics **(B–D)** during strides with different initial foot contacts. **(A)** Superimposed foot trajectories across all strides and all neonates (top), ensemble-averaged (mean ± SD) vertical foot displacements vs. normalized gait cycle (bottom), and the amplitude of vertical foot movements (mean ± SD). As the relative duration of stance varied across strides, a white region indicates an amount of variability in the stance phase duration. **(B)** Corresponding joint angular movements and their ROMs. **(C)** Ensemble-averaged foot, thigh and shank elevation angles and examples of 3-dimensional gait loops and interpolation planes on the bottom (4 steps for each condition). Gait loops are obtained by plotting the thigh waveform vs. the shank and foot waveforms (after mean values subtraction). The interpolation planes result from orthogonal planar regression. Percentage of total variation explained by 1st, 2nd, and 3rd principal components (PV_1_, PV_2_, and PV_3_, respectively) is shown on the right. **(D)** Spherical spatial density of the u_3_ vector directions for all steps. Each point corresponds to a single stride. Color scale indicates density diagrams calculated using the Kamb method for axial data with *E* = 2σ and exponential smoothing (see methods), the black contours outline the areas with density equal to *E* + 2σ. Asterisks denote significant differences between conditions.

Figure 5 illustrates the temporal characteristics of limb motion. In all conditions, the foot path was variable and tended to have only one peak during the swing phase. The foot lift was significantly higher in the heel contact condition [ANOVA *F*_(2, 186)_ = 6.13 *p* = 0.003, Tukey tests *p* ≤ 0.005, Figure 5A]. The range of motion of the proximal segments and joint angles was similar (Figures 5B,C). Interestingly, there was a significant effect of condition on the ROM in the distal ankle joint [ANOVA *F*_(2, 186)_ = 7.25 *p* = 0.0009], being greater for steps with the initial forefoot contact with respect to steps with the midfoot (*p* = 0.008, Tukey HSD) and heel (*p* = 0.002) foot contacts. The difference in the ankle joint ROM between heel and forefoot step conditions was ~20°.

The inter-segmental coordination (Figures 5C,D) was assessed using the principal component analysis of limb segment elevation angles (see Methods). Figure 5C illustrates ensemble-averaged thigh, shank, and foot elevation angles. Despite inter-stride variability, temporal changes of the elevation angles of lower limb segments covaried along a plane, describing a characteristic loop over each stride (see examples in the lower panels of Figure 5C). Paths progress in time in the counter-clockwise direction along the loop, touchdown and lift-off corresponding approximately to the top and bottom of the loop, respectively. Planarity was quantified by the percentage of variance accounted for by the third eigenvector (PV_3_) of the data covariance matrix (for ideal planarity PV_3_ = 0% and the third eigenvalue = 0). In all conditions, PV_3_ was small (on average 2–3%, Figure 3C, right panel). The percentage of variance accounted for by the second eigenvector (PV_2_) depended on the condition [ANOVA *F*_(2, 181)_ = 5.29 *p* = 0.006] and it was significantly smaller during steps with the initial heel (16 ± 9%) and midfoot (19 ± 10%) contact than in steps with heel contacts (23 ± 10%, *p* ≤ 0.03 Tukey tests), indicating a wider gait loop during strides with the initial forefoot contact. The orientation of the covariance plane (normal to the plane, u_3_) was variable across strides (Figure 5D, left color plots) reflecting generally high kinematic variability of neonate stepping. The orientation of the plane was nevertheless slightly but significantly different across conditions, in particular, the u3_s_ parameter [ANOVA *F*_(2, 181)_ = 6.61 *p* = 0.002, Tukey tests *p* ≤ 0.01, Figure 5D, right panel].

In sum, since the gait loop and its associated plane depend both on the amplitude and phase of the limb segment oscillations, the observed differences (PV_2_ and u_3_) in the planar covariation pattern confirm the general effect of the initial foot contact on the kinematics of the ongoing step (Figures 3, 4).

### EMG patterns during steps with different initial foot contacts

**Figure 6:**
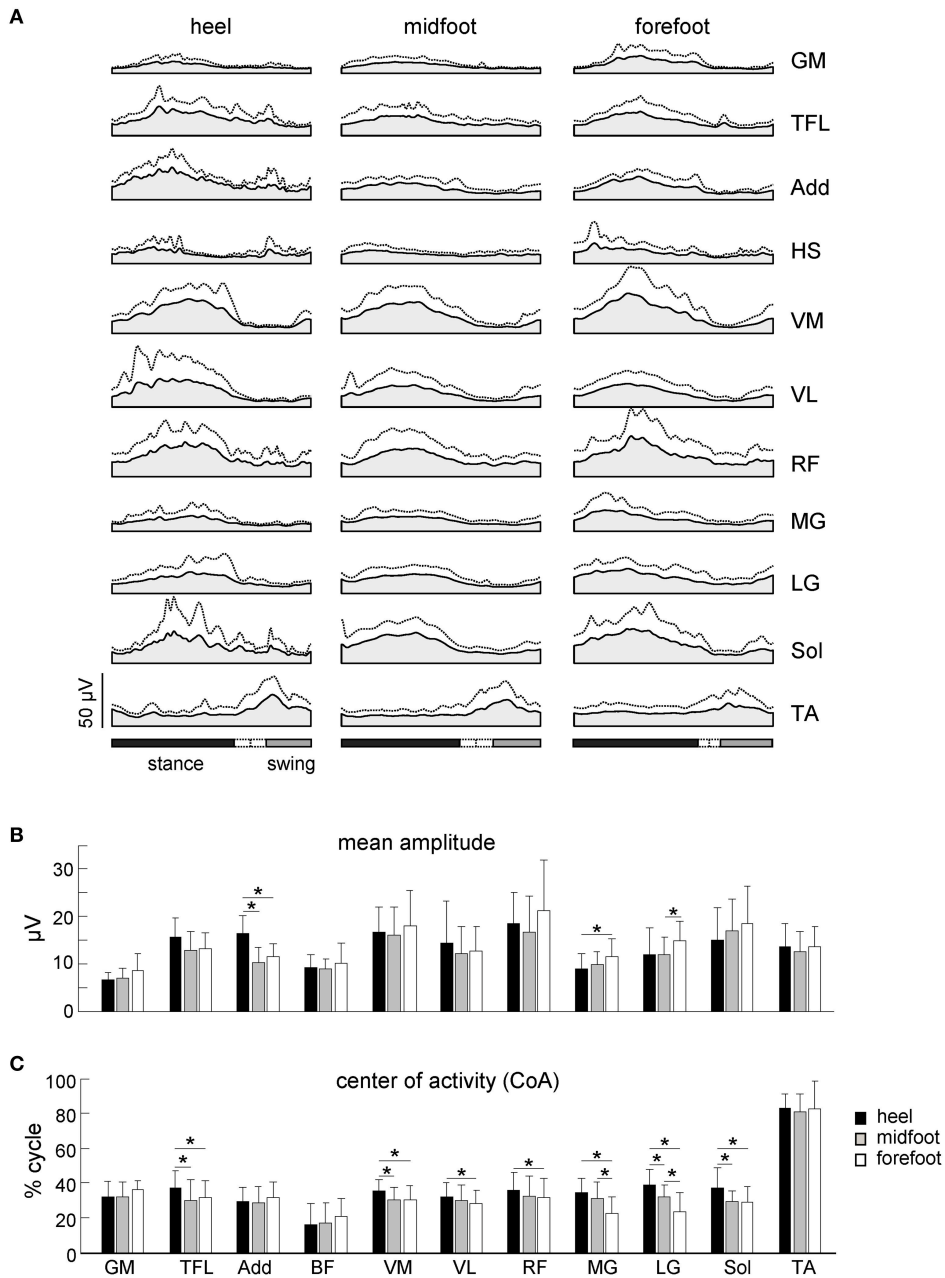
Characteristics of EMG activity. **(A)** Ensemble-averaged (mean + SD) EMG activity patterns of 11 ipsilateral leg muscles plotted vs. normalized gait cycle. All strides with different initial foot contacts for all neonates were pooled together. **(B)** Mean leg muscle EMGs (means + SD). **(C)** Center of activity (CoA). Asterisks denote significant differences between conditions. Note a shift of the CoA in most muscles to the later stance for the heel contact condition.

Figure 6A shows ensemble-averaged EMG activity profiles of 11 ipsilateral (to the foot contact) leg muscles. Despite variability in time-varying structure of muscle activation both between infants and across step cycles, this figure reflects the general features of neonate stepping responses. Many of the leg muscles of the infant are co-activated over the stance phase with alternate left/right coordination, except for TA, which is mostly active during swing (Forssberg, 1985; Yang et al., 1998; Okamoto et al., 2001; Dominici et al., 2011; Teulier et al., 2012). As a result, the EMG activity profiles of most muscles showed the peak around midstance and a lack of activity around the double support phase (Figure 6A), consistent with previous reports. The duration of the activations was generally long, lasting about one-half of the cycle, which is in marked contrast with the adult pattern of relatively brief activations occurring primarily at touchdown and lift-off even under body weight support conditions (Ivanenko et al., 2002).

While the mean amplitude of muscle activity (Figure 6B), EMG profiles (Figure 6A) and general characteristics of steps (cycle duration, stance duration, step length and range of motion of the proximal joints, Figures 4A, 5B) were similar, the initial foot contact significantly correlated with the center-of-activity in both distal and proximal extensors (MG, LG, Sol, VM, VL, RF, TFL, Watson-Williams tests, *p* < 0.049 for all muscles, Figure 6C). On average, the CoA of extensor muscles was shifted toward the end of the stance phase by ~5% of the gait cycle in the “heel” condition with respect to the forefoot condition, which corresponds to about 170 ms (given that the mean cycle duration was ~3.5 s, Figure 4A).

## Discussion

In the current study, we aimed to classify the neonatal steps in relation to the initial foot contact (Figure 1) in order to describe the detailed characteristics of foot placements, plantar pressure distribution and their possible relation to motor patterns. The results demonstrated inter-individual changes in the foot placement and load distribution underneath the plantar surface of the foot associated with different kinematic and muscle activity patterns (Figures 3–6), suggesting an expression of a variety of footfall patterns and the potential role of contact- or load-related proprioceptive feedback already during early manifestations of stepping movements on ground in human babies.

### Characteristics of the footfall and motor patterns during stepping on ground in neonates

The general stepping parameters (cycle duration, step length, range of motion of proximal joints) were similar across conditions of foot contact (Figure 4A). Nevertheless, the initial foot contact significantly affected the center-of-pressure excursion (Figure 3C), and correlated with the range of motion in the ankle joint (Figure 5B) and the center-of-activity of extensor muscles (being shifted by ~5% of cycle toward the end of stance in the “heel” relative to “forefoot” condition, Figure 6C). Given the wide distribution of gait cycle duration in neonates (Yang et al., 2015), this shift could not be accounted for by the effect of speed, since the cycle duration and stepping speed were similar across conditions (Figure 4A) and since the normalized EMG activity profiles in neonates do not vary much with cycle duration (Ivanenko et al., 2013). More likely, temporal changes in the muscle activity and limb motion dynamics were related to the specificity in the control of different footfall patterns.

The three major footfall patterns were identified with the initial heel, midfoot and forefoot contacts, respectively. The initial heel touchdown was observed in about 25% of steps (Figure 1B), thus it is present already very early during development, consistent with the pioneering observations by Forssberg (1985). It is important to note, however, that these steps in neonates differ from the adult heel-to-toe roll-over in the dynamics of ground reaction forces and associated muscle activity patterns (as well as the flexed posture of the newborn). Even when the infants demonstrated a heel “strike,” the “adult-like” two peaks in the vertical ground reaction force at weight acceptance and propulsion (Figure 3D, left panel) and EMG bursts accompanying these sub-phases of stance (Figure 6A) were normally lacking. It is also worth noting that the two-peaked profile and muscle activity around the step-to-step transition in adults are present even when the body weight is partially unloaded (Ivanenko et al., 2002). Rather, podobarografic and extensor muscle activity recordings showed typically one peak around midstance, consistent with tiny shear forces (Forssberg, 1985) and the lack of EMG activity around the double support phase in neonates (Dominici et al., 2011). Analogously, steps with the initial forefoot contact differed from the digitigrade gait in animals or prominent toe-walking in children with cerebral palsy since the foot did not remain attached to the ground only by forefoot during the stance phase. The CoP could migrate toward the heel area (Figure 2C), as well as the range of motion in the ankle joint significantly increased in this condition (Figure 5B).

Asymmetries in leg movements might be present in newborn stepping responses (Domellöf et al., 2007), but we limited our kinematic analysis to the right (recorded) leg and its specific initial foot contact. The analysis of the intersegmental coordination revealed subtle but significant differences in the spatiotemporal kinematic patterns between initial foot contact conditions (Figure 5). Interestingly, similar to adults and older children (Dominici et al., 2007), temporal changes of the elevation angles of lower limb segments covary along a plane (Figure 5C), describing a characteristic loop over each stride. However, despite variability inherent to neonatal stepping (Figure 5D), there was a systematic trend in the planar covariation parameters depending on the initial foot contact. Since the gait loop and its associated plane depend both on the amplitude and phase of the limb segment oscillations, the observed differences (PV_2_ and u_3_) in the planar covariation pattern (Figures 5C,D) confirm the general effect of the initial foot contact on loading and the kinematics of the ongoing step (Figures 3, 4).

The schematic Figure 4C illustrates major kinematic differences between steps with initial heel and forefoot contacts. In the heel contact condition, the limb angle at touchdown and lift-off tended to be larger (more flexed limb), the ankle joint being more dorsiflexed, the toes more flexed and the foot more lifted during swing. If one considers that the flexion reflex may be a part of the stepping mechanism (Sherrington, 1910; Duysens et al., 2013), steps with the initial heel contact were characterized by an increased flexion of the hip, ankle and toes (Figure 4B).

We do not know the mechanisms underlying manifestation of different footfall patterns in neonates, though several factors can account for it. These may include immature descending pathways and maturational changes in sensory input (Yakovlev and Lecours, 1967; Eyre et al., 2001; Martin, 2005; Yeo et al., 2014; Yang et al., 2015). Peripheral factors may also contribute to the sensorimotor integration. For instance, even in adults there are notable foot deformations caused by foot soft tissue deformations (Wright et al., 2012), which should be much more prominent in infants due to a fat pad underneath the foot plantar surface. Slower contraction time of muscles (Dayanidhi et al., 2013), maturation of nerve conduction time (attributable to increasing fiber diameter; Eyre et al., 1991) and ossification of the foot skeleton (Maier, 1961) also affect the peripheral shaping of the locomotor output. Moreover, the influence of the supraspinal inputs on the functioning and outcome of the spinal pattern generation circuitry cannot be ruled out (Barbu-Roth et al., 2014; Anderson et al., 2016; Ritterband-Rosenbaum et al., 2017).

## Limitations of the current study

When interpreting the current results, one should take into account the following limitations. Even though the stepping pattern is relatively immune from descending brain control during the first year of life (Yang et al., 2015), the level of arousal mediated by the supraspinal structures is essential for newborn infant stepping (Thelen et al., 1982), so that individual changes in excitability of the spinal circuitry or in muscle tone may influence foot motion characteristics. For instance, Barbu-Roth et al. (2009, 2015) have argued that newborn stepping may be more open to descending control than prior research would suggest. Thus, additional measurements (EEG, fNIRS, etc.) might help to clarify the influence of supraspinal control on spinal pattern generators (Lacquaniti et al., 2017; Ritterband-Rosenbaum et al., 2017).

Body weight support was provided manually and its variation could contribute to the variation in foot placement. Although the stepping reflex is a stereotyped leg movement that can be evoked in newborns when held upright and inclined forward with the feet touching the ground (Zelazo et al., 1972; Forssberg, 1985; Yang et al., 2004; Dominici et al., 2011; Minassian et al., 2017), some oscillations in the up-down and lateral forces and trunk displacements can be expected when manually maintaining an upright position. Moreover, even though we did not analyze the kinematic patterns bilaterally, some asymmetries between leg movements were previously documented in newborn babies (Domellöf et al., 2007). On the other hand, we did not find significant side differences in the ground force parameters. The infants were allowed to support as much of their own weight as possible, the rest being supported by a hospital pediatrician. Also, four pediatricians performed these experiments, and they might have held the newborns somewhat differently. Yet, the mean level of the BWS estimated from the recorded maximal ground reaction force (Figure 3D) was similar across pediatricians, and importantly all three types of the initial foot contact were present independently of the pediatrician holding the infant. It is also unlikely that the amount of vertical ground floor forces represent the major source of the observed footfalls (Figure 1A) since the differences in the estimated level of body weight support were on average small across conditions (<4% between heel and forefoot contacts, Figure 3D right panel). Nevertheless, some influences of the manual BWS on the foot placement characteristics cannot be excluded.

Finally, based on the experimental design, it is not possible to determine whether the initial foot contact affects the motor patterns or whether the initial foot contact is an expression (reflection) of a motor pattern that has been pre-selected and pre-parameterized. Further experiments are needed to clarify the contribution of central and peripheral factors.

## Conclusion

In summary, we found a variety of initial foot contacts along with associated changes in the motor patterns during stepping on ground in neonates. These findings suggest the potential contribution of load-related proprioceptive feedback and/or expression of variations in the locomotor program already during early pattern generation circuitry development in human babies. In addition to investigating the basic mechanisms of gait maturation, there is growing interest in quantifying dynamic, muscle activity and kinematic patterns and functional asymmetries in infants as predictive indicators of impaired motor development (Fallang and Hadders-Algra, 2005; Domellöf et al., 2007; Barbu-Roth et al., 2009; Kanemaru et al., 2014; Ritterband-Rosenbaum et al., 2017). Therefore, such studies may be worthwhile and necessary in clinical environments for the assessment and early diagnosis of infants with motor impairments, which can be facilitated by a thorough knowledge of infant's foot function maturation.

## Author contributions

Conceived and designed the experiments: YI, ADP, PP, and FL. Performed the experiments: GC, AF, SP, ADP, PP, FL, and YI. Analyzed the data: FSL, SM, GC, and VL. Wrote the paper: FSL, FL, and YI. All the authors made contributions in drafting the manuscript and interpreting the results and have approved the final version.

### Conflict of interest statement

The authors declare that the research was conducted in the absence of any commercial or financial relationships that could be construed as a potential conflict of interest.
