# Corrigendum: Foot Placement Characteristics and Plantar Pressure Distribution Patterns during Stepping on Ground in Neonates

**DOI:** 10.3389/fphys.2017.00973

**Published:** 2017-11-27

**Authors:** F. Sylos-Labini, S. Magnani, G. Cappellini, V. La Scaleia, A. Fabiano, S. Picone, P. Paolillo, A. Di Paolo, F. Lacquaniti, Y. Ivanenko

**Affiliations:** ^1^Center of Space BioMedicine, University of Rome Tor Vergata, Rome, Italy; ^2^Neuromotor Physiology Laboratory, Fondazione Santa Lucia (IRCCS), Rome, Italy; ^3^Department of Computer, Control and Management Engineering, Sapienza University of Rome, Rome, Italy; ^4^Neonatology and Neonatal Intensive Care Unit, Casilino Hospital, Rome, Italy; ^5^Neonatology and Neonatal Intensive Care Unit, Ospedale San Giovanni, Rome, Italy; ^6^Department of Systems Medicine, University of Rome Tor Vergata, Rome, Italy

**Keywords:** neonates, stepping pattern, limb loading, foot pressures, kinematics, EMG activity, early development, human locomotion

In the original article, we neglected to include the specific section **“Acknowledgments”** in the article. We would like to add to the article:

## Conflict of interest statement

The authors declare that the research was conducted in the absence of any commercial or financial relationships that could be construed as a potential conflict of interest.

